# The Diverse and Dynamic Nature of *Leishmania* Parasitophorous Vacuoles Studied by Multidimensional Imaging

**DOI:** 10.1371/journal.pntd.0001518

**Published:** 2012-02-14

**Authors:** Fernando Real, Renato A. Mortara

**Affiliations:** Department of Microbiology, Immunology and Parasitology, Escola Paulista de Medicina, UNIFESP, São Paulo, Brazil; Institut Pasteur, France

## Abstract

An important area in the cell biology of intracellular parasitism is the customization of parasitophorous vacuoles (PVs) by prokaryotic or eukaryotic intracellular microorganisms. We were curious to compare PV biogenesis in primary mouse bone marrow-derived macrophages exposed to carefully prepared amastigotes of either *Leishmania major* or *L. amazonensis*. While tight-fitting PVs are housing one or two *L. major* amastigotes, giant PVs are housing many *L. amazonensis* amastigotes. In this study, using multidimensional imaging of live cells, we compare and characterize the PV biogenesis/remodeling of macrophages i) hosting amastigotes of either *L. major* or *L. amazonensis* and ii) loaded with Lysotracker, a lysosomotropic fluorescent probe. Three dynamic features of *Leishmania* amastigote-hosting PVs are documented: they range from i) entry of Lysotracker transients within tight-fitting, fission-prone *L. major* amastigote-housing PVs; ii) the decrease in the number of macrophage acidic vesicles during the *L. major* PV fission or *L. amazonensis* PV enlargement; to iii) the *L. amazonensis* PV remodeling after homotypic fusion. The high content information of multidimensional images allowed the updating of our understanding of the *Leishmania* species-specific differences in PV biogenesis/remodeling and could be useful for the study of other intracellular microorganisms.

## Author Summary

*Leishmania* parasites lodge in host cells within phagolysosome-like structures called parasitophorous vacuoles (PVs). Depending on the species, amastigote forms can be individually hosted within small, tight-fitting PVs or grouped within loose, spacious PVs. Using multidimensional live cell imaging, we examined the biogenesis of the two PV phenotypes in macrophages exposed to *L. major* (a representative of the tight PV phenotype) or *L. amazonensis* (an example of the loose PV phenotype) amastigotes. *L. major* PVs undergo fission as parasites divide; we demonstrate that in the course of fission there are transients of the lysosomotropic fluorescent probe Lysotracker. In contrast, during the course of amastigote population size expansion, *L. amazonensis* PVs do accumulate Lysotracker while increasing in diameter and volume. The large PVs fuse together, and the products of fusion undergo size and shape remodeling. The biogenesis/remodeling of the two types of *Leishmania* PVs is accompanied by a reduction in the number of macrophage acidic vesicles. The present imaging study adds new morphometric information to the cell biology of *Leishmania* amastigote intracellular parasitism.

## Introduction

*Leishmania* spp. are dimorphic trypanosomatid parasites that alternate between extracellular promastigote forms found in insect vectors and intracellular amastigote forms found in mammalian hosts. In infected cells, *Leishmania* amastigotes are sheltered within phagolysosome-like structures called parasitophorous vacuoles (PVs). The PV membranes and contents change as PVs fuse with the endoplasmic reticulum (ER), late endosomes, lysosomes, or other host cell vesicular elements, conferring to them distinctive properties and a hybrid nature [1]–[3].

In the majority of *Leishmania* species, including *L. major*, one or two amastigotes are enclosed within PVs, which display a modest vacuolar space. In contrast, the large PVs that shelter parasites of the *L. mexicana* complex, such as *L. amazonensis*, can contain numerous amastigotes, often bound by their posterior poles to the internal face of the PVs [4]. The biogenesis of these two types of PVs involves the acquisition of host cell late endosomes membrane markers, as shown in infected cells immunostained for lysosome-associated membrane proteins (LAMPs), Rab GTPases, cathepsin, proton ATPases, and MHC class II molecules [2], [5]–[8]. The acquisition of these markers is a coordinated event that results in a “mature” PV, which is presumably required for the survival and multiplication of the parasites.

Because the relatively large dimensions of their PVs that allow them to be easily recognized at low magnification, *L. amazonensis* and *L. mexicana* PVs have often been used in studies of the fusogenic properties of *Leishmania* PVs. These PVs could be demonstrated to selectively fuse with each other or with phagosomes containing macromolecules, colloids, inert particles, and other live parasitic microorganisms [9]–[17]. The fusogenicity and easy access of certain particles and molecules to these large structures increase with the duration of infection [1]. The spacious PVs also incorporate acidic pH markers such as Lysotracker and neutral red [17], [18] and may be probed using pH-sensitive dyes [19], [20]. By taking advantage of differences in fluorescein emission under different pH conditions, it was reported that the pH of *L. amazonensis* PVs falls from approximately 5.2 at 24 h to 4.8 after 48 h of intracellular infection, whereas the pH of secondary lysosomes of around 5.4 remained constant in non-infected control cells [19].

These studies led to the characterization of the biochemical and functional features of *Leishmania* PVs, which may not apply to the majority of *Leishmania* species studied that are lodged in tight PVs and assumed to undergo fission as parasites divide [21], [22]. The accessibility of particles, macromolecules, and probes to these tight-fitting PVs and the identification of their contents are hindered by the limited vacuolar space available between the parasites and their PV membranes. Chang and Dwyer [21] and Berman and colleagues [23] observed by electron microscopy that thorium dioxide (“Thorotrast”) particles, which were pre-loaded in lysosomes, were transferred to the small vacuolar space of *L. donovani* and *L. major* PVs, respectively. The granules were absent within PVs when parasites and PV membranes were only in close contact. These studies suggest that tight-fitting *Leishmania* PVs can fuse with lysosomes, although the retention of lysosomal markers differs accordingly to PV dimensions. Additionally, the pH in tight-fitting PVs may be different from that within loose vacuoles: the pH within *L. donovani* tight PVs was reported to reach 5.5 after 2 h of infection [24].

Most of the available information on *Leishmania* PV biogenesis has been obtained by experiments on fixed cells, a drawback we sought to overcome in the present study. We examined the biogenesis of large or tight-fitting, membrane-bound *Leishmania* PVs recorded by the multidimensional imaging of live infected macrophages. The fission of *Leishmania* tight-fitting PVs was studied for the first time in live infected cells and characterized as a two-step process that involves the replication of amastigotes in a single PV prior to separation into two distinct PVs that accumulate transient amounts of lysosomotropic probe. The process is accompanied by the depletion of macrophage small acidic compartments, as previously described for *Leishmania* large vacuoles [25]. The biogenesis of these large structures was also studied and revealed to involve PV enlargement in volume and diameter to the detriment of other PVs in the same infected cells. The homotypic PV fusion between *L. amazonensis* PVs was recorded and involves PV volume restoration.

## Materials and Methods

### Ethics statement

All experiments involving animal work were conducted under Brazilian National Commitee on Ethics in Research (CONEP) and French National Committee on Ethics and Animal Experimentation (CNREEA) ethic guidelines, which are in accordance with international standards (CIOMS/OMS, 1985). The present study was approved by CEP/UNIFESP (Comitê de Ética em Pesquisa da Universidade Federal de São Paulo/Hospital São Paulo) under the protocol number 0856/07.

### Host cells and parasites

BALB/c and BALB/c nude mice (8 weeks of age) were used as sources of bone marrow macrophage precursor cells and lesion-derived *Leishmania* amastigotes. Macrophages were obtained from bone marrow precursor cell suspensions cultivated in vitro for 7 days in RPMI 1640 medium with 10% fetal calf serum, 5% L929 cell conditioned medium, 100 U/ml penicillin, and 100 µg/ml streptomycin [7]. RAW 264.7 macrophage-like cells were cotransfected with LAMP1-GFP and Rab7-GFP plasmids (using FuGene HD transfection reagent, ROCHE) kindly donated by Dr. Norma Andrews (Maryland University) and employed in infection experiments in order to observe PV membranes in live recordings.

Macrophages were transferred to glass coverslips or round dishes (ibidi, GmbH or Mattek Corporation) suitable for maintaining living cells in incubators coupled to microscopes. Before their use in experiments, cultures were incubated overnight at 37°C in a humidified air atmosphere containing 5% CO_2_.

BALB/c nude mice footpads were inoculated with wild-type *L. (L.) amazonensis* LV79 (MPRO/BR/72/M1841) or DsRed2-transfected *L. (L.) major* NIH173 (MHOM/IR/-/173). Isolation of amastigotes from footpad lesions was performed as previously described [26] after 2 months of inoculation.

### Infection of macrophage cultures

*Leishmania* amastigotes were added to macrophage cultures at a multiplicity of infection of 5 and incubated at 34°C and 5% CO_2_ in complete medium for different periods according to the experiment. Cultures were washed with Hanks' Buffered Salt Solution to remove free parasites and cultivated in complete medium at 34°C in a 5% CO_2_ atmosphere. Observation and image acquisition of live or fixed macrophage cultures under the employed microscopes started after periods ranging from 2 to 48 h depending on the experiment. Cultures were maintained at 34°C and 5% CO_2_ within the incubators coupled to the microscopes.

### Immunolabeling of *Leishmania* PVs

Macrophages on coverslips were washed and fixed for 1 h with 3.5% formaldehyde in phosphate-buffered saline (PBS). *Leishmania* PVs and other compartments were identified by immunolabeling of the membrane proteins LAMP1 and LAMP2 (monoclonal antibodies obtained from DSHB, Iowa University, USA). *L. amazonensis* amastigotes were immunostained with 2A3-26 antibody conjugated to FITC (kindly provided by Dr. Eric Prina, Institut Pasteur, France) or loaded with 5 µM 5,6-carboxyfluorescein diacetate succinimidyl ester (CFSE, Invitrogen, Life Technologies). Samples were stained for 15 min with 100 µg/ml 4′,6-diamidino-2-phenylindole (DAPI, Invitrogen, Life Technologies) and mounted with 50% glycerol in PBS containing 0.01% *p*-phenylenediamine. Confocal images were obtained using a Bio-Rad 1024 UV system coupled to a Zeiss Axiovert 100 microscope or a Leica TCS SP5 II system. Images acquired with a 100× (1.44 NA) oil immersion objective were rendered with Imaris Software (Bitplane AG) by using Blend filters.

### Acquisition of multidimensional images

Live imaging of cultures was performed using a Nikon Biostation IM-Q Live cell recorder system (Nikon Corporation), a Perkin-Elmer UltraView RS Nipkow-disk system (PerkinElmer Inc.) attached to a Zeiss Axiovert 200 M microscope with a Hamamatsu ORCA II ER CCD camera, or in a Leica TCS SP5 II system (Leica Microsystems). To identify *Leishmania* PVs, 50 nM Lysotracker green DND-26 (Invitrogen, Life Technologies), a lysosomotropic probe for acidic compartments, was added to complete medium 1 h before microscopic recordings and maintained throughout image acquisition. In contrast with *L. amazonensis* PVs that were rich in Lysotracker, vacuoles containing a single *L. major*-DsRed2 parasite displayed a feeble Lysotracker signal, possibly due to the lower acidity of their PVs. The effect of a tight vacuolar space between PV membranes and amastigotes on Lysotracker intensity is not discarded, although Lysotracker is a relatively small molecule (MW = 398.69). Alternatively, 1 mg/ml FITC-dextran (average mol wt 42,000, Sigma-Aldrich) was used as lysosomotropic probe, with a 1 hour pulse, removal of the probe by 6 washings prior to image acquisition.

The Nikon Biostation IM-Q was used to acquire, in 10 different microscopic fields, serial images of infected macrophage cultures in dishes. The Biostation acquired images in phase contrast and in two fluorescent channels (for Lysotracker- and DsRed2-labeled parasites) with a 40× (0.8 NA) objective in 5-min intervals. Points in time of time-lapse image acquisitions are displayed as day, hours, and minutes (d:hh:mm). Images of the DsRed2 fluorescence signal displayed by *L. major* were processed by Acapella software (PerkinElmer Inc.) for algorithm-based quantification of these parasites during infection in macrophage cultures [17].

The Perkin-Elmer UltraView RS and the Leica TCS SP5 II system were used to acquire stacks of 20 to 30 optical sections from live infected cells in 5 to 12 microscopic fields. Stacks along the z- axis (z-stacks) were obtained with an optical section separation (z-interval) of 0.2 to 1 µm.

### Multidimensional imaging software analysis

We acquired images of infected cell cultures after different post-infection times. Some image acquisitions began 2 h after parasite addition to macrophages; other started after 24 h or 48 h. Thus, we chose to present temporal data as “time of image acquisition” instead of “time of infection” due to different time ranges of intracellular infection. The time of multidimensional acquisition is displayed as d:hh:mm.

#### Measurement of parasites and PV features using isosurfaces

Acquired z-stacks were reconstructed into multidimensional image projections by using Imaris software (version 7.3.1×64, Bitplane AG, Zurich, Switzerland). Blend or MIP filters were used for tridimensional visualization. Imaris creates isosurface/isospot objects by filtering the original data set and overlaying mathematical models over the original data. The software reports measurement statistics based on the set of voxels (pixels with z dimension) that are completely inside isosurface/isospot objects. The attribution of isosurface/isospot objects was based on the Lysotracker or DsRed2 fluorescent channels, which enclosed voxels with RFIs (Relative Fluorescence Intensities) as given by the acquisition system (in arbitrary units). The mean and minimum RFI measurements were associated with the results as indicators of fluorescence quality and reliability of isosurface/isospot detection; the mean RFI can be associated with biological variations in fluorescence intensities (i.e. Lysotracker RFI fluctuations in different vacuolar pHs), whereas minimum RFIs represent the set of voxels with lower fluorescence signals and can be taken as a quality control for the fluorescence signal. Temporal data were discarded when microscopic fields display decrease in Lysotracker minimum or sum of RFI measurements. Mean, minimum and sum are standard mathematical functions on voxel analysis.

Isosurface rendering was used to measure volume, diameter, and RFI from an identified structure. Isosurfaces corresponding to *L. major*-DsRed2 amastigotes were constructed from DsRed2 fluorescence signals, enabling the parameters of background subtraction and split touching object (seed detection diameter of 2.5 µm). Although they were constructed from the DsRed2 fluorescence channel, *L. major* amastigote isosurfaces enclosed Lysotracker voxels, allowing the measurement of Lysotracker RFIs associated with the regions in which amastigotes are located.

Isosurfaces corresponding to Lysotracker clusters associated to *L. major* amastigotes were constructed with background subtraction parameter enabled; for the spacious vacuoles formed by *L. amazonensis*, isosurfaces were attributed using the enabled background subtraction and split touching objects parameters (seed detection diameter of 5–10 µm). *Leishmania* PV isosurfaces were colorized according to their volumetric measurements, with blue-cyan representing smaller volumes and purple-pink representing larger volumes. Vacuole diameters were measured by examination of the intermediate optical sections of each sample.

The accuracy of software volume measurements was tested by comparing *L. major*-DsRed2 amastigote volumes measured by the software and the volume calculated assuming that amastigotes are ellipsoid geometric structures/bodies. The formula for ellipsoid volume is 4/3πxyz, where x is the width axis radius, y is the length axis radius, and z is the height axis radius. The software volumetric measurements were compatible with ellipsoid forms with radii near 2, 2.5, and 2, respectively.

#### Macrophage acidic vesicles detection and quantification using isospots

Multidimensional images of macrophages infected with *L. amazonensis* or *L. major*-DsRed2 and non-infected macrophages were separately acquired in the same experiments using multi-chamber dishes (ibidi Hi-Q4, GmbH). Lysotracker was kept in complete medium throughout the experiments and images were obtained using the same acquisition parameters. Using Imaris software, acidic vesicles were recognized as circular structures of 0.5 or 1 µm in diameter with a specified RFI threshold (parameters were adjusted in the first time point of each multidimensional image). To avoid the identification of acidic vesicles from vicinal macrophages, the available cell identification tool was used. Although stained with the same fluorescent marker, the RFI quantitative method can distinguish the different RFI values of small, dispersed vesicles from those of large *Leishmania* PVs, allowing separate software analysis for these two different compartments.

### Statistics

All statistics were performed using SPSS software (SPSS Inc.). The statistical tests employed are indicated in the figure legends, and they were chosen on the basis of normal and non-normal distributions and equal and non-equal variances. Figures represent the same result reproduced in at least 2/3 out of all analyzed multidimensional images acquired in 3 different experiments, using 2 different equipments.

## Results

### The *L. amazonensis* parasitophorous vacuoles

#### Development of the spacious *L. amazonensis* PVs

**Figure 1:**
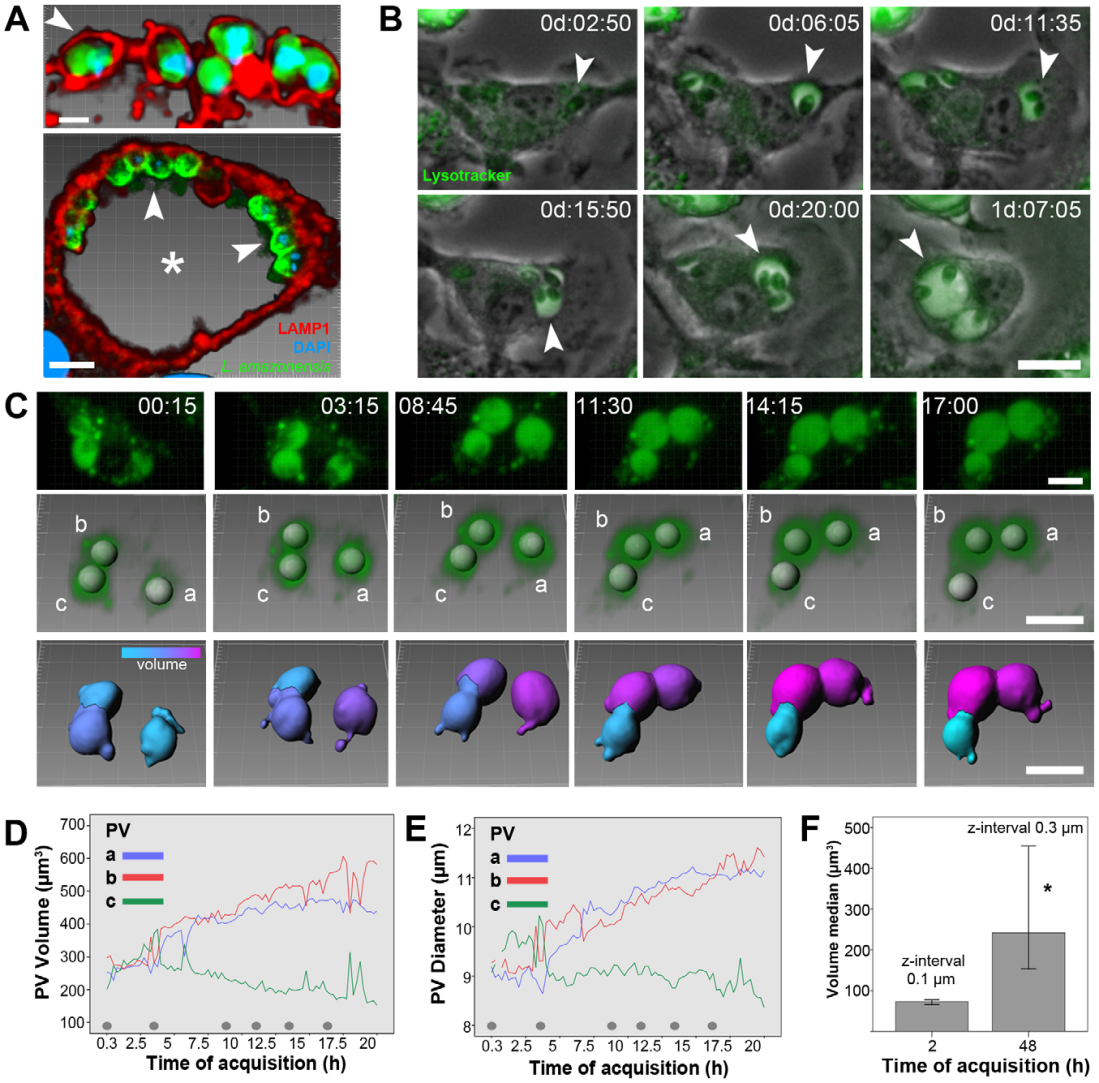
Enlargement of *L. amazonensis* PVs. (A) Immunostaining of LAMP1 for *L. amazonensis* PV identification in fixed samples of infected macrophages. Red color represents LAMP1 labeling, blue color represents DAPI stain, and green color represents CFSE staining (upper panel) or immunostaining with *L. amazonensis*-specific antibody (lower panel). Arrowheads indicate parasites sheltered within tight-fitting PVs after 2 h of infection (upper image, bar = 2 µm) and within large PVs sheltering several amastigotes after 48 h of infection (lower image, bar = 5 µm). (B) Expansion of *L. amazonensis* PVs recorded by time-lapse microscopy of infected macrophage cultures loaded with Lysotracker (green color). Arrowhead indicates an enlarging PV from a tight-fitting phenotype to a large, communal, Lysotracker-positive phenotype. Time-lapse acquisition started after 2 h of infection, and the time of acquisition is shown as d:hh:mm. Bar = 10 µm. (C) Multidimensional imaging of *L. amazonensis* PVs loaded with Lysotracker in live infected macrophages. The first row of images shows Lysotracker signals exhibited by three large PVs during the recordings. Multidimensional acquisition started after 2 h of infection, and the time of acquisition is shown as hh:mm. The second row shows the software recognition of the three PVs, represented by tridimensional objects; for each object, the software attributed an isosurface (a, b, and c), which permitted measurements of PV volume, diameter, and RFI. In the third row, isosurfaces a, b, and c, representative of each PV, display a statistic-coded color in accordance with volume measurements ranging from cyan (smaller volume) to magenta (larger volume). Bar = 10 µm. (D–E) The isosurface measurements of volume (D) and diameter (E) are shown; although isosurfaces objects a and b increased in volume and diameter, isosurface c presented a discreet decrease in its dimensions. Gray dots in the graphs indicate the time points to which images presented in C are associated. The graphs are an example of 20 macrophage multidimensional images in which PVs enlarged in size. (F) The volumes of *L. amazonensis* PVs were measured after 2 and 48 h of intracellular infection using multidimensional images acquired using 0.1- or 0.3-µm z-intervals, respectively. The graph shows the median PV volumes measured from three different microscopic fields for each time point (median ± confidence interval, n = 3). There is a significant (P<0.05) increase in PV volumes (Wilcoxon Signed Rank Test), and the measurements were compatible with those acquired using multidimensional images constructed from a z-interval of 1 µm.

The growth of *Leishmania* PVs was followed by sequential microscopic imaging of macrophages infected with *L. amazonensis* amastigotes (Figs. 1–3). Amastigotes were initially lodged in tight-fitting LAMP1-positive PVs in the first hours of infection (Fig. 1A, upper image, 2 h post-infection). These PVs increased in size (reaching diameters of the order of 10 µm) and maintained the LAMP1-positive phenotype, with apparently higher concentrations of this lysosome marker in the contact sites between PV membranes and parasite posterior poles (Fig. 1A, lower image, 48 h post-infection). Time-lapse recordings of macrophages infected with *L. amazonensis* in the presence of the fluorescence lysosomotropic probe Lysotracker revealed the development of PVs from the tight-fitting phenotype (around 1 h post-infection) to the enlarged, Lysotracker-positive phenotype (Fig. 1B and Video S1-A). Time-lapse recordings after 96 h of infection showed fully developed *L. amazonensis* PVs with moderate PV enlargement (Video S1-B), suggesting a stationary phase in the growth of these structures.

**Figure 2 pntd-0001518-g002:**
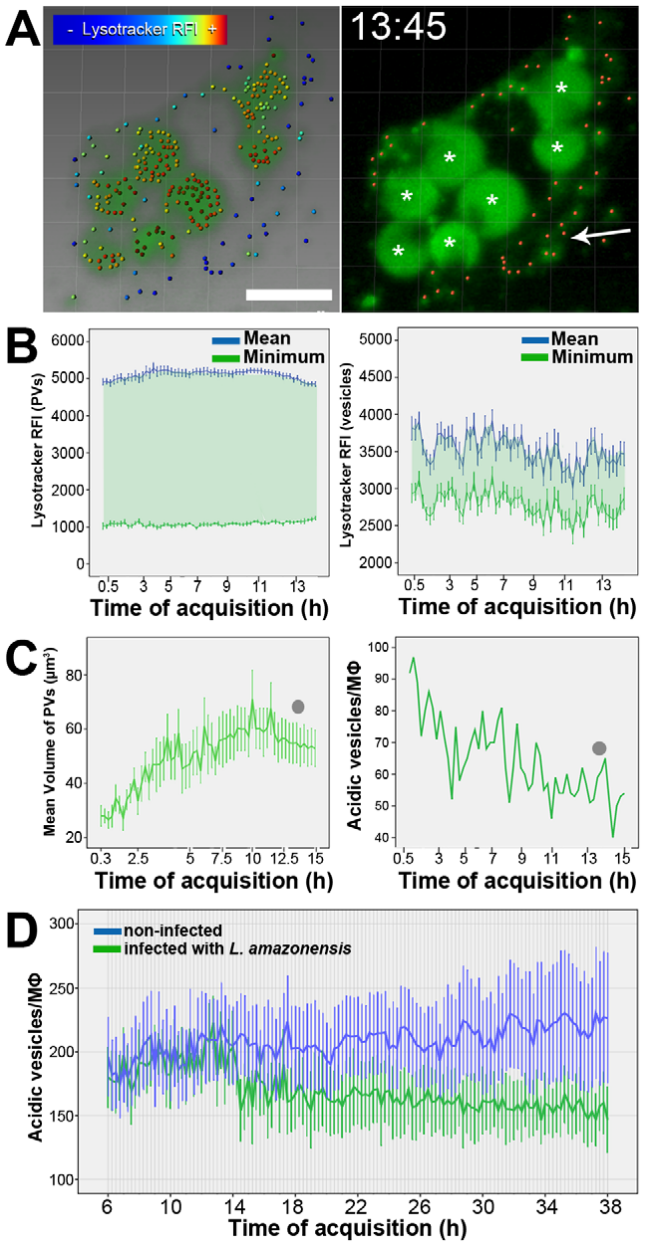
Macrophage acidic vesicles decrease in number during *L. amazonensis* PV growth. (A) Separated identification of acidic vesicles and *Leishmania* PVs using the same fluorescence channel. In the left image, isospots 1 µm in diameter were attributed by the software to Lysotracker channel voxels of multidimensional images. The software identified small vesicles surrounding *L. amazonensis* PVs but interpreted PVs as clusters of Lysotracker-positive vesicles. Each isospot has a statistic-based color corresponding to the Lysotracker RFI mean value (colored bar). By adjusting the thresholds of isospot detection based on Lysotracker RFI, the software can attribute isospots for the acidic vesicles (orange isospots in the second image, indicated by arrow), excluding the fluorescence signal of *Leishmania* PVs (asterisks). Blend and MIP filters, bar = 10 µm. Image acquisition started after 2 h of *L. amazonensis* infection, and the time of acquisition is shown (hh:mm). (B) Lysotracker RFIs from *L. amazonensis* PVs measured using isosurfaces (graph in the left) and Lysotracker RFIs from acidic vesicles measured using isospots (graph in the right) in the same multidimensional image (mean ± SEM, n = 7 [for PVs], n = 40−80 [for vesicles]). (C) *L. amazonensis* PVs increase in volume (graph in the left, mean ± SEM, n = 7), whereas macrophage acidic vesicles decrease in number (graph in the right) in the same macrophage. Gray dots in the graphs indicate the time points to which images presented in A are associated. (D) Quantification of the number of detected acidic vesicles (1 µm in diameter) in macrophages infected with *L. amazonensis* (green line) and non-infected macrophages (blue line). Images of these two conditions were separately acquired in the same experiment using multi-chamber dishes with the same acquisition parameters. Data are representative of 8 infected macrophages and 12 non-infected macrophages (mean and SEM) and reproduced in two experiments.

**Figure 3:**
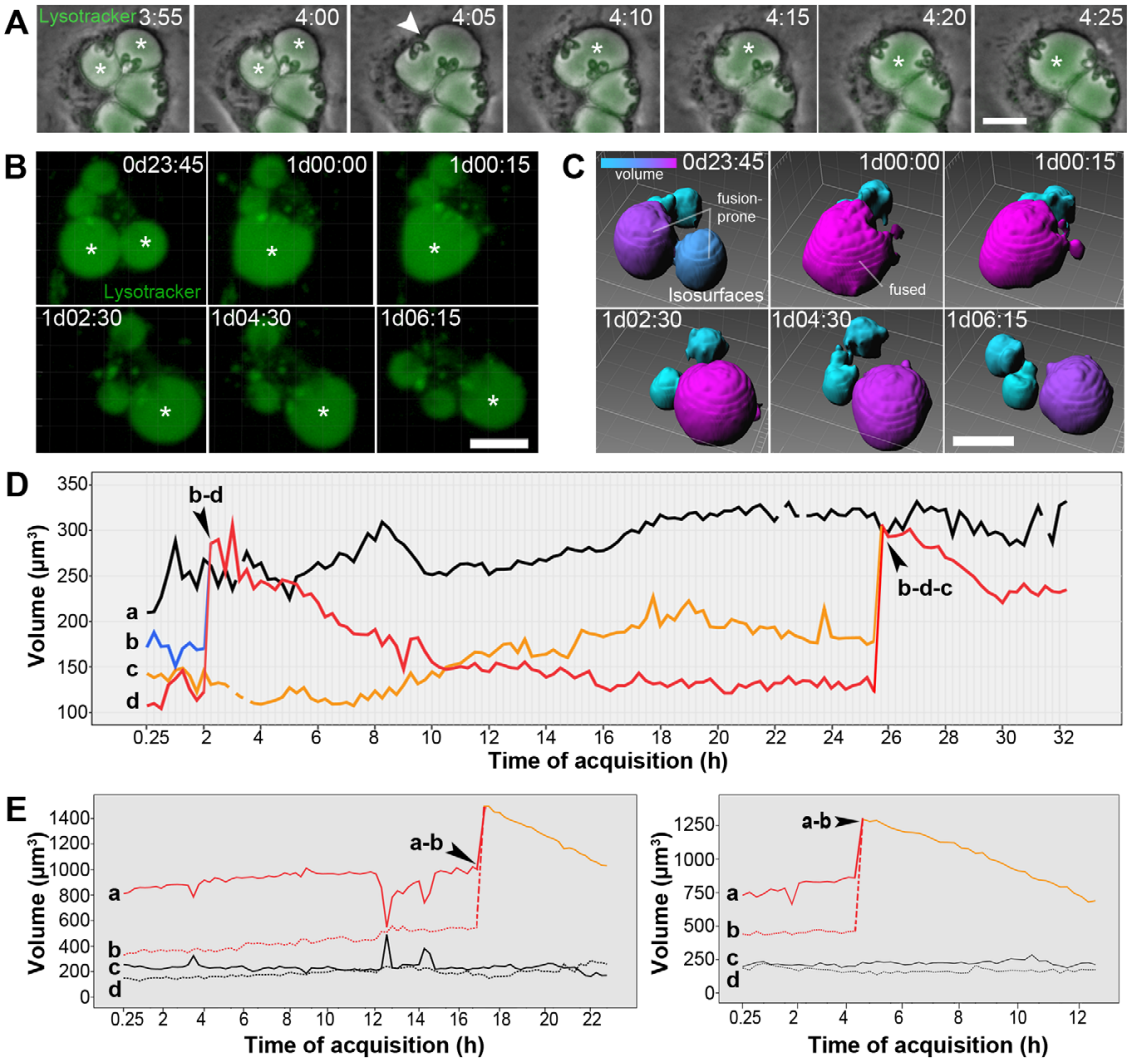
Fusion and remodeling of *L. amazonensis* PVs. (A) Fusion between *L. amazonensis* PVs recorded by time-lapse microscopy of infected macrophage cultures loaded with Lysotracker (green). Asterisks indicate PVs involved in fusion; arrowheads indicate an intermediary PV morphology following membrane fusion. Time-lapse acquisition started after 48 h of infection, and the time of acquisition is shown as h:mm. Bar = 10 µm. (B–C) Multidimensional imaging of *L. amazonensis* PVs loaded with Lysotracker in live infected macrophages. In B, Lysotracker fluorescence of an infected macrophage containing four PVs. Asterisks indicate the PVs involved in fusion. In C, the isosurfaces representative of each PV in the multidimensional image. Isosurfaces have statistic-based color according to their measured volumes ranging from cyan (smaller volume) to magenta (larger volume). Fusion-prone PVs and fused vacuole are indicated. Image acquisition started after 48 h of infection, and the time of acquisition is shown as d:hh:mm. Bar = 10 µm. (D) Volume measurements of *L. amazonensis* PVs (a–d) in an infected macrophage (data acquisition started from 2 h of infection). Fusion between PVs is indicated by arrowheads, and the PVs involved in fusion are identified (i.e., when b fused with d, b–d). (E) Volume measurements of *L. amazonensis* PVs (a–d) in two different infected macrophages (data acquisition started from 48 h of infection). Fusion between PVs is indicated by arrowheads, and the PVs involved in fusion are identified. Another 5 multidimensional images of fusion-prone PVs were also analyzed and presented the same results.

The strong Lysotracker fluorescence signal displayed by *L. amazonensis* PVs permitted the measurement of PV volume and diameter throughout intracellular infection (Figs. 1C–E and Video S2). The analysis software used can process the sample focal stacks at different time points (Fig. 1C, first row) into tridimensional objects, recognize *L. amazonensis* PVs, and attribute to them an isosurface object with volume and diameter information (Fig. 1C, isosurfaces a, b, and c, second row). The constructed isosurfaces in the third row represent each *L. amazonensis* PV that were colorized according to their measured volumes.

Measurements of PV volume for each time point are plotted in Figs. 1D–E. The increase in PV volume/diameter occurred while other *L. amazonensis* PVs maintained their dimensions. In the first 20 h of image acquisition, PVs doubled in volume (Fig. 1D), whereas their diameters increased by approximately 20% (Fig. 1E). A plateau in volumetric measurements was observed for isosurfaces after approximately 12 h of image acquisition (Fig. 1D).

The median volume values of PVs from different microscopic fields were reproduced using shorter z-stack intervals, which are more accurate for volumetric measurements (Fig. 1F). The PV volume measurements acquired after 2 h or 48 h of *L. amazonensis* infection and using 0.1-µm or 0.3-µm z-stack intervals yielded volume measurements comparable to those presented in Fig. 1D.

#### The number of host cell acidic vesicles decreases over the course of *L. amazonensis* infection

We next examined whether *L. amazonensis* PV enlargement could be associated with the classically described depletion of the host cell secondary lysosomes, assumed to be one of the main membrane sources for *L. amazonensis* PV biogenesis [25]. In the present experiments, we estimated the number of small (up to 1.0 µm in diameter) Lysotracker-positive vesicles in uninfected macrophages or those infected with *L. amazonensis* amastigotes.

Fig. 2A and Video S3 show the Lysotracker thresholding procedure that can be used to distinguish *Leishmania* PVs from macrophage acidic vesicles. The attribution of isosurfaces (for PVs) and isospots (for vesicles) used different Lysotracker signal thresholds. Thus, from an image containing all Lysotracker clusters (Fig. 2A, image on the left) in which isospots detect different Lysotracker relative fluorescence intensities (RFIs), it was possible to select those isospots with a specific Lysotracker pattern that corresponded to macrophage acidic vesicles (Fig. 2A, image on the right). Graphs of Figure 2B show the mean and minimum Lysotracker RFIs detected by isosurfaces (attributed to PVs, graph on the left) and isospots (attributed to vesicles, graph on the right), confirming the different thresholds in which the detection of acidic vesicles was based.

Using the attributed isosurfaces and isospots, PV volumes and the numbers of macrophage acidic vesicles in infected cells were estimated (Fig. 2C, right panel). Extending classic qualitative observations, we found that the number of small acidic vesicles decreased as the PV mean volume increased in infected macrophages.

This approach was applied to microscopic fields containing infected or non-infected macrophages, separated by multi-chamber culture dishes. The graph presented in Fig. 2D shows the quantification of acidic vesicles in these two examined groups of macrophages. Over the course of image acquisition, the number of acidic vesicles in the infected macrophages decreased in comparison with non-infected macrophages. These results indicate that *L. amazonensis* PV enlargement is correlated with a decrease in the host cell acidic vesicles reservoir.

#### *L. amazonensis* PVs recover their dimensions after homotypic fusion

Membrane input to *L. amazonensis* PVs is also provided by the homotypic fusion between these vacuoles [16]. In the present studies, fusion events between *L. amazonensis* PVs in infected macrophages were recorded by time-lapse and multidimensional microscopy (Fig. 3 and Video S4). Acquisitions started after 48 h of intracellular infection, when PVs are sufficiently enlarged to contact and fuse [16]. In fluorescence time-lapse microscopy, a 5-min interval between images permitted the observation of fusion between *L. amazonensis* PVs with temporal resolution (Fig. 3A, asterisks). The fused PV exhibited an intermediary shape compatible with the hemifusion stage of membrane fusion events (Fig. 3A, arrowhead) and recovered its circular diameter in a few minutes.

To measure PV volume fluctuations during and after fusion, images were acquired from infected cells displaying recognizable PV fusion events; isosurfaces were attributed to each Lysotracker-positive PV (Figs. 3B–C and Video S4). Fig. 3B shows the multidimensional image of four *L. amazonensis* PVs in the same infected macrophage loaded with Lysotracker. Fusion between PVs (indicated by asterisks) was captured. The isosurfaces constructed from the Lysotracker signal displayed by these PVs, were colorized according to their measured volumes and shown in Fig. 3C. Fusion between *L. amazonensis* PVs yielded a larger vacuole, which unexpectedly recovered its initial dimensions 6 h after homotypic fusion.

This result was also observed in other infected cells (Figs. 3D–E). PV volume measurements at different time points were plotted in the graph in which *L. amazonensis* PVs are represented by isosurfaces named a–d. Fusion-prone PVs with similar initial volumes resulted in a 2-fold larger fused PV. However, after fusion, PV dimensions approached the initial volume measurements displayed by the vacuoles involved in fusion. This result was observed in successive fusion events in the same infected cell (Fig. 3D, with image acquisition started after 2 h of infection) and also in other recorded fusion events in different macrophages infected for different time periods (Fig. 3E, image acquisition started after 48 h of infection).

### The *L. major* Parasitophorous Vacuoles

#### Division of *L. major* amastigotes within tight-fitting PVs

**Figure 4:**
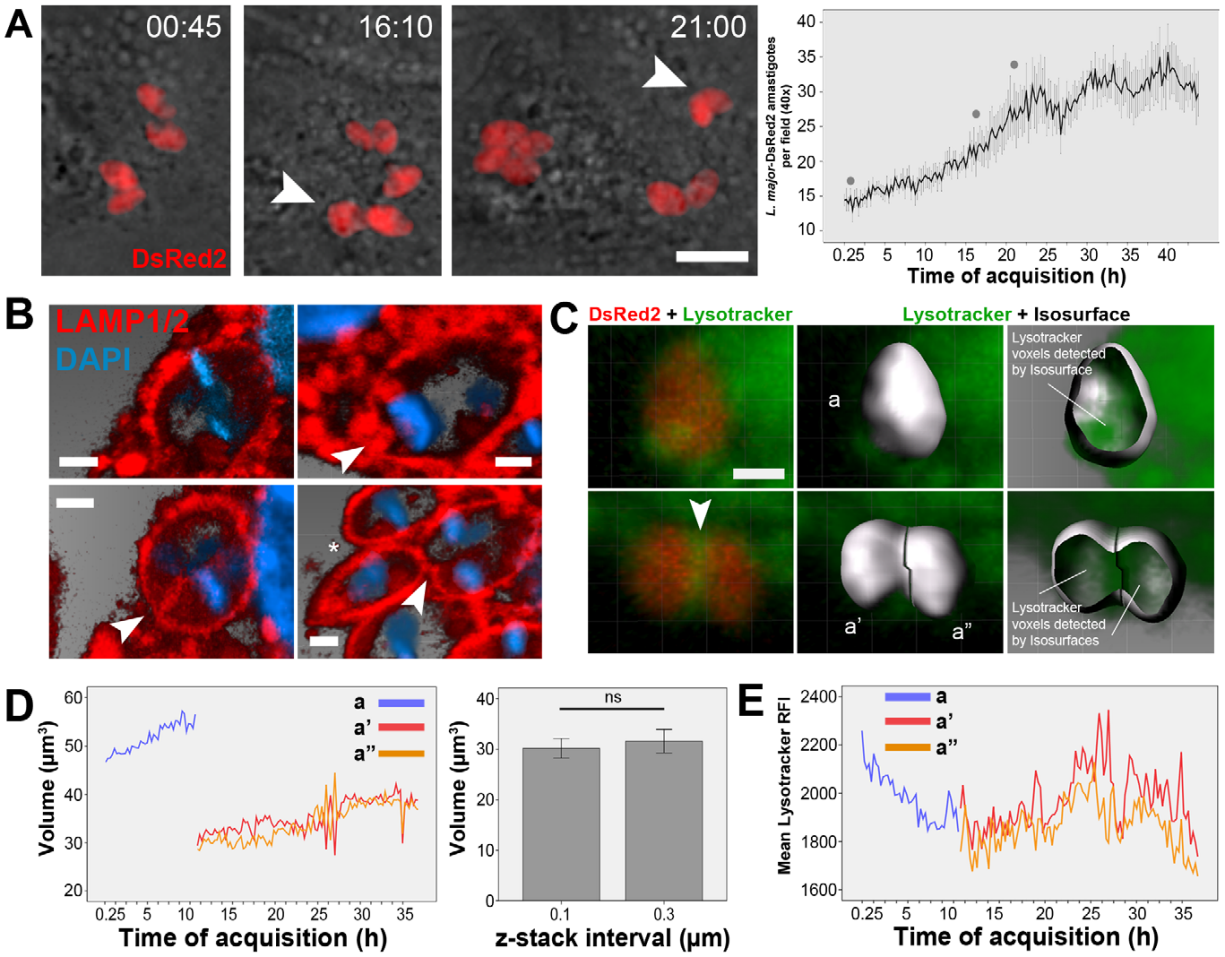
Replication of *L. major* amastigotes in tight-fitting PVs. (A) Multiplication of *L. major*-DsRed2 amastigotes recorded by fluorescent time-lapse microscopy of infected macrophage cultures. Arrowheads indicate dividing parasites (red fluorescence) that were located near each other for several hours. Time-lapse acquisition started after 2 h of infection, and the time of acquisition is shown as hh:mm. Phase contrast merged with DsRed2 fluorescence (red). Bar = 10 µm. Parasites were quantified per microscopic field (40× objective) by algorithm-based recognition of DsRed2 fluorescence, expressed by amastigotes (mean ± SEM, n = 10). The record comprises the period in which the parasite population doubles. Gray dots in the graph indicate the time points to which images are associated. (B) Immunostaining of LAMP1 and LAMP2 proteins for *L. major* PV identification in fixed samples of macrophages infected with *L. major* amastigotes for 48 h. Red represents LAMP1/LAMP2 immunostaining, and blue represents DAPI staining. Image shows amastigotes during division process sharing the same PV which presented polarized fission furrows (arrowheads). Asterisk show completely separated amastigotes in individualized PVs. Blend filter, bars = 1 µm. (C) Multidimensional imaging of *L. major*-DsRed2 (red) amastigotes hosted by live macrophages loaded with Lysotracker (green). First column: Lysotracker weakly stains individual tight-fitting PVs. During division, a Lysotracker cluster in the interface between two dividing *L. major* amastigotes is observed in some cases; MIP filter. Using the DsRed2 signal expressed by amastigotes (second column, Blend filter), isosurfaces were constructed representing the parasites before (a) and after division (a′ and a″), shown in the third column. The Lysotracker voxels can be detected by the isosurfaces (with transparency applied). Bar = 2 µm. (D) Volume measurements of *L. majo*r-DsRed2 amastigotes from isosurfaces in multidimensional images. A volumetric increase in isosurface a (before division) was detected, followed by division into isosurfaces a′ and a″, which present half of the initial value of isosurface a (line graph, in the left). The amastigotes volumes were also measured in other multidimensional images with z-stack intervals of 0.1 and 0.3 µm (bar graph, mean ± SEM, n = 3) with non-significant (ns) statistical difference (one-way ANOVA). (E) Mean Lysotracker RFI values detected in *L. major* isosurfaces before (a) and after parasite division (a′ and a″). Values diverge between divided isosurfaces a few hours hours after their identification. Results shown in D–E were reproduced in 5 other multidimensional images in which *L. major* amastigote divisions and lysotracker-positive interface were identified.

In the following observations we recorded the behavior of *L. major* tight-fitting PVs during parasite division in infected macrophages by time-lapse fluorescence microscopy and multidimensional imaging (Figs. 4–6). The algorithm-based quantification of *L. major*-DsRed2 amastigotes hosted by macrophages over a 48-h period is shown in Fig. 4A. The time sequence in figure 4A and Video S5 shows four *L. major*-DsRed2 amastigotes hosted by a macrophage that divide into eight amastigotes sheltered in non-observable PVs in the course of the recordings. After 12 h of acquisition, dividing *L. major*-DsRed2 amastigotes were often observed (arrowhead), and they remained next to each other for several hours before complete separation occurred. The numbers of *L. major*-DsRed2 amastigotes per field were plotted in the graph on the right: the population of amastigotes doubles in 24 h of image acquisition, which began after 2 h of *L. major*-DsRed2 infection. In macrophages fixed after 48 h of infection, the immunostaining for the PV membrane components LAMP1 and LAMP2 indicates that *L. major* PVs containing two or three amastigotes (some of them undergoing binary division) may be observed (Fig. 4B). PV membrane furrows are often observed in these double occupancy PVs (arrowheads in Fig. 4B) near amastigote anterior pole. These results suggest that parasite division within PVs precedes *L. major* PV fission.

**Figure 5 pntd-0001518-g005:**
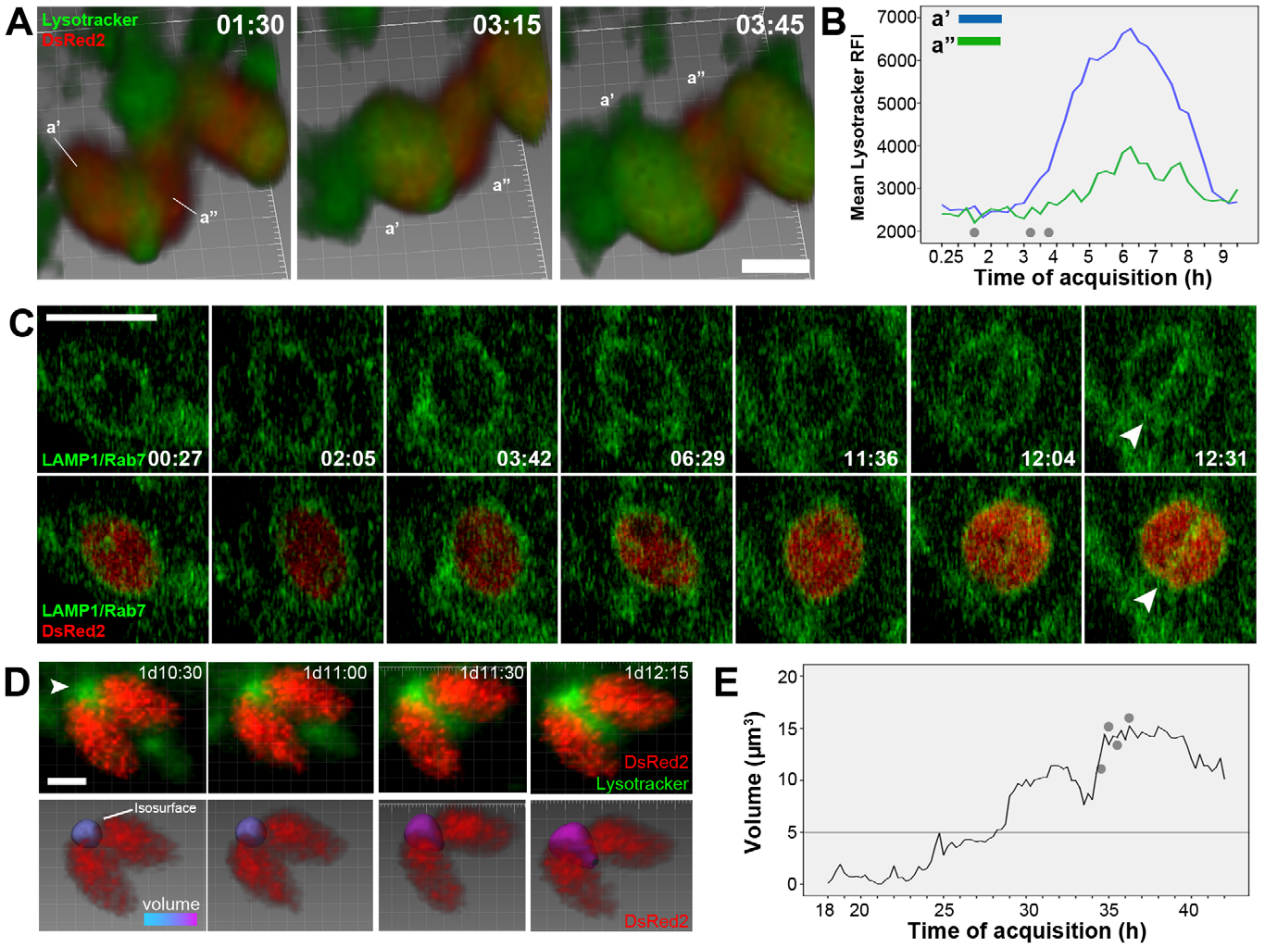
Fission of *L. major* PVs. (A) Multidimensional image of dividing *L. major*-DsRed2 amastigotes (red) hosted by macrophages loaded with Lysotracker (green); a lysotracker-positive interface between the parasites is clearly observed at 1:30h. Image acquisition started after 48 h of infection, and the time of acquisition is shown as hh:mm. Blend filter, bar = 2 µm. Isosurfaces a′ and a″ were attributed to replicating amastigotes using the DsRed2 channel. (B) The Lysotracker RFIs surrounding parasites was measured during 10 h of image acquisition. An increase in the Lysotracker signal was detected for one of the dividing amastigotes, whereas the signal remained low in the other amastigote. After 8 h of acquisition, although both amastigotes remained next to each other, they were surrounded by Lysotracker at low intensities, and no Lysotracker-positive vacuolar interface was observed between them. Gray dots in the graphs indicate the time points to which images presented in E are associated. The data are representative of 5 cases with similar results. (C) Multidimensional image of RAW 264.7 macrophages expressing LAMP1-GFP and Rab7-GFP (green) infected with *L. major*-DsRed2 (red). Arrowheads indicate the complete division of double-occupancy PV into two individual PVs. This process was followed in 5 other multidimensional images and the time period in which amastigotes share a single vacuole before fission was about 100 minutes. Acquisition started after 4 h of intracellular infection and the time of acquisition is shown as hh:mm. MIP filter, bar = 5 µm. (D) The Lysotracker-positive cluster is observed between two dividing parasites (first row, MIP filter). The attribution of an isosurface to the Lysotracker-positive interface (second row, Blend filter) allowed the measurement of its volume at the image time points. The isosurface contains statistic-based color information ranging from cyan (smaller volumes) to magenta (larger volumes). Image acquisition started after 2 h of infection, and the time of acquisition is shown as hh:mm. Bar = 5 µm. (E) The graph presents the volume measured from the cluster between dividing parasites in the multidimensional imaging. Using a z-stack interval of 1 µm, a detection limit for volumetric measurement of this structure was determined to be 5 µm^3^. Gray dots in the graphs indicate the time points to which images presented in D are associated. These results were reproduced in 5 other cases.

**Figure 6:**
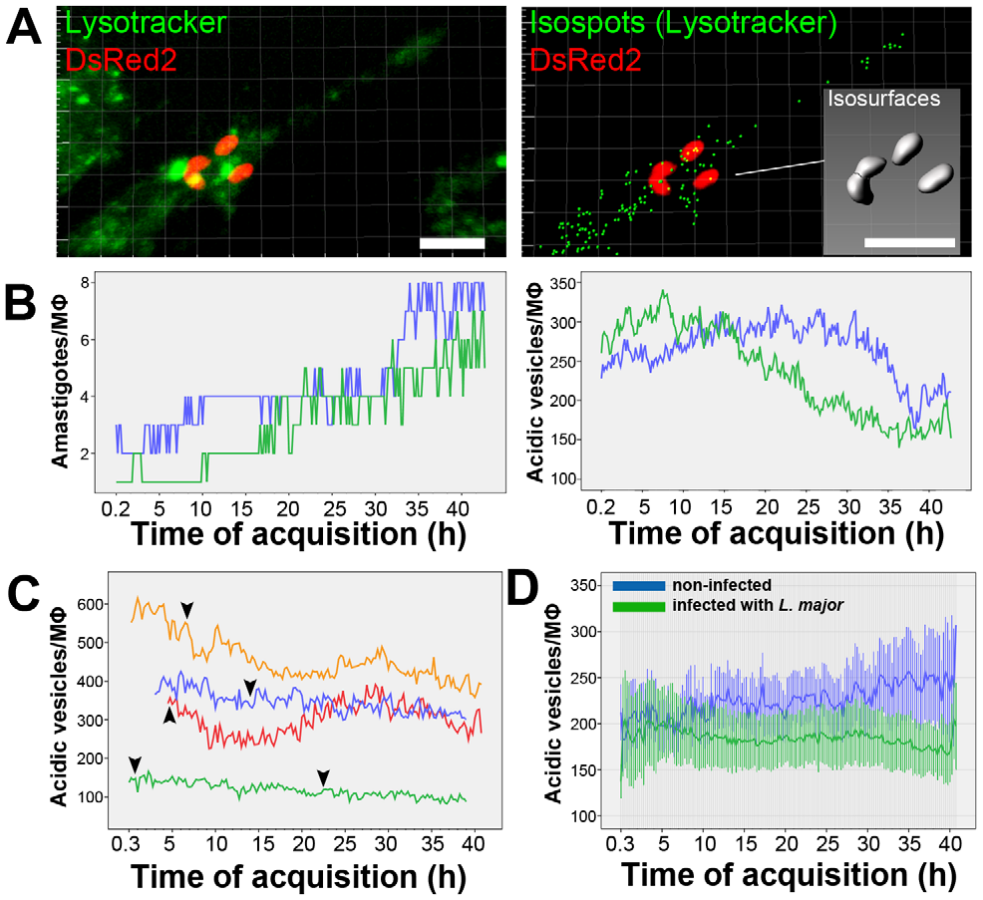
Numerical decrease of host cells acidic vesicles during *L. major* division. (A) Multidimensional imaging of a macrophage loaded with Lysotracker and hosting *L.* major-DsRed2 replicating amastigotes after 48 h of infection. On the left, Lysotracker (green) and DsRed2 (red) merged signals, MIP filter. On the right, isospots were attributed by software for macrophage acidic vesicles (green) considering Lysotracker fluorescence intensity; isosurfaces for parasites (constructed from DsRed2 fluorescence intensity) were also attributed (in the detail, gray). Bar = 10 µm. (B) Quantification of the number of amastigotes detected isosurfaces and the number of detected acidic vesicles (1 µm in diameter) in two macrophages (green and blue lines). (C) Quantification of the number of detected acidic vesicles (1 µm in diameter) in other four macrophages (lines colored accordingly) - arrowheads indicate the moments when at least one amastigote began to replicate. (D) Mean number of detected acidic vesicles in macrophages infected with *L. major*-DsRed2 (green line) and non-infected macrophages (blue line). Images of these two conditions were separately acquired in the same experiment using multi-chamber dishes, and the same acquisition parameters. Data are representative of 14 infected macrophages and 12 infected macrophages (mean and SEM) and reproduced in two experiments.

To detect single- or double-occupancy tight-fitting PVs and their dynamics during parasite division in live recordings, infected macrophages were loaded with Lysotracker after infection (Fig. 4C). An amastigote is presented before (upper row) and after its division (lower row), when Lysotracker may concentrates in the interface between dividing amastigotes (Video S5).

Analytical isosurfaces were attributed for each parasite considering the DsRed2 signal (a, before division; a′ and a″, after division). The isosurfaces contain information on amastigote dimensions and the Lysotracker RFIs in their surroundings (representative of the probe concentration within tight-fitting PVs), and these measurements were plotted in Figs. 4D–E. The *L. major*-DsRed2 isosurface increased in volume before division in two other recognizable isosurfaces, each with approximately half of its original volume (Fig. 4D, first graph). *L. major*-DsRed2 amastigotes presented a volume of approximately 30 µm^3^, measured from multidimensional images using z-stack intervals of 0.1 or 0.3 µm, as shown by the bar graph. Fig. 4E shows the Lysotracker RFIs around replicating *L. major*-DsRed2 amastigotes. Amastigotes exhibited higher Lysotracker RFI before division, which is associated to a polarized concentration of the dye probably related to the parasite flagellar pocket. In the first hours post-division, amastigotes present an intermediate Lysotracker RFI (Fig. 4E, after 12 h), sometimes associated to a clusterization of the probe between two dividing amastigotes.

At the beginning of image acquisition, these Lysotracker RFIs are 1.6 times lower than the intensities found in phagosomes containing aldehyde-fixed *L. major*-DsRed2 parasites (Video S5). Similar results were obtained when FITC-dextran was used as lysosomotropic probe instead of Lysotracker (Video S5). The higher concentration of Lysotracker or FITC-dextran enclosed within fixed amastigote phagosomes indicates that live *L. major* amastigotes customize their PVs and restrict the access of late phagosome content markers.

#### Fission of *L. major* PVs

Fission of *L. major* PVs was inferred from differences in Lysotracker RFI retained in fissioned vacuoles and directly observed by staining PV membranes with fluorescent phagolysosomal markers. Figure 5A and Video S6 show *L. major*-DsRed2 amastigotes during division, displaying the previously described Lysotracker cluster. At early time points of acquisition, the vacuolar interface remains between amastigotes in which isosurfaces (a′ and a″) detect the approximate values of Lysotracker RFI (as shown by the associated graph, Fig. 5B). After 3 h of acquisition, an increase in Lysotracker RFI was measured surrounding one parasite (a′), whereas the measurements were constant for the other parasite (a″). At the end of image acquisition, although the amastigotes remained close to each other, no Lysotracker-positive interface could be detected and the Lysotracker RFI surrounding amastigotes returned to the initial values.

*L. major*-DsRed2 PV fission was also observed in infected RAW 264.7 macrophage-like cells expressing LAMP1 and Rab7 proteins tagged with GFP (Fig. 5C and Video S6). In this experiment, PV membrane could be observed during the fission process: *L. major*-DsRed2 dividing amastigotes are confined to phagolysosomal tight-fitting vacuoles during the entire formation of new PVs, from single occupancy, passing through double occupancy and finally to segregation in two structures. The interval between division of the amastigote and PV complete fission was registered in these multidimensional images, and periods range from 84 to 153 minutes (108.8±11.8 SEM., n = 5).

The Lysotracker-positive interface observed between dividing amastigotes appears to be associated with PV double occupancy. During amastigote division, a Lysotracker cluster may be detected between some division-prone parasites, suggestive of a vacuolar space in double-occupancy PVs or a stable interaction site between PVs and acidic vesicles (Fig. 5D, arrowhead, and Video S7). This intense Lysotracker signal between parasites was tracked in multidimensional images, and its volume measured at each acquisition time point using isosurfaces for the Lysotracker signal. The cluster presented a dynamic volume, as shown by the volume-based color of the attributed isosurface (Fig. 5D, second row). Volume was plotted at all acquisition time points (Fig. 5E) and increased as macrophage acidic vesicles interacts with the analyzed structure (Video S7).

The results thus suggest that after sharing the same PV, dividing *L. major*-DsRed2 amastigotes were sheltered in different PVs that differed in the amounts of Lysotracker they contained, although preserving phagolysosomal membrane markers.

#### Host cell acidic vesicles and *L. major* infection

Considering the phagolysosome-like features of *Leishmania* PVs, it may be expected that the partition of doubly occupied *L. major*-DsRed2 PVs would require the recruitment of different membrane sources such as small acidic vesicles to the sites of dividing parasites. To determine whether new tight-fitting PVs could be formed while acidic vesicles are depleted, acidic vesicles (1 µm in diameter) were separately scored in the multidimensional images of macrophages infected with *L. major*. Fig. 6A shows a multidimensional image of an infected macrophage hosting four *L. major*-DsRed2 amastigotes derived from the replication of two amastigotes (Video S8). As described previously, the software attributed isospots for macrophage acidic vesicles (Fig. 6A, on the right) and analytical isosurfaces for *L. major*-DsRed2 amastigotes (Fig. 6A, insert), both tracked at different time points (Video S8).

The quantification of macrophage acidic vesicles during *L. major*-DsRed2 infection is shown in parallel with the quantification of parasite numbers in the same macrophage (Fig. 6B, two macrophages analyzed). The numerical decrease of acidic vesicles correlates in time with *L. major* multiplication. Figure 6C shows four other cases in which this decrease was also quantified. Arrowheads in the graph indicate the time points when an amastigote division began. The mean number of acidic vesicles detected in non-infected and infected macrophages was plotted in Fig. 6D. These two conditions were acquired separately in multi-chamber dishes in the same experiment, using the same acquisition parameters. Quantifications yielded similar amounts of acidic vesicles in the first 10 h of image acquisition; from this moment, macrophages infected with *L. major* amastigotes presented a decrease in the mean number of acidic vesicles. This decrease is not so evident than the decrease found in case-case analysis because of the lack of synchronicity of *L. major* divisions detected in different cells.

## Discussion

PVs are the host intracellular compartments in which *Leishmania* parasites differentiate and multiply. They are lined by a dynamic membrane originated at the host cell plasma membrane and formed by successive and coordinated fusion/fission events with vesicles from early/late endocytic pathways, secondary lysosomes, ER, and possibly autophagic vesicles, resulting in an acidic compartment similar to phagolysosomes [3], [5], [7], [19], [27]. Most of this information has been obtained in experiments with *Leishmania* cell-cycling amastigotes of the *L. mexicana* group in fixed preparations.

This study presents high-resolution morphological characterization of the main features displayed by *Leishmania* PVs in live infected macrophages, such as PV volumetric expansion/retraction and homotypic PV fusion/fission, using the fluorescent lysosomotropic probe Lysotracker. Additionally, a software-based methodology permitted the quantification of host cell acidic vesicles, which were distinguished from *Leishmania* PVs in the same fluorescence channel. As secondary lysosomes are considered the main source of membrane for *Leishmania* PV biogenesis, we investigated how macrophage acidic vesicles reservoirs (which include, but are not restricted to secondary lysosomes) were related to *Leishmania* PV biogenesis.

The development of spacious PVs is an exception rather than a rule for most *Leishmania* parasites studied. Soon after the internalization of *L. amazonensis*, PV membranes acquire Rab5 and EEA1, two early endosomes markers; by membrane remodeling, PV membranes lose these early markers and rapidly acquire the late endosome and lysosome markers Rab7p and LAMP1 [7]. In the first hours of intracellular infection, fusion between PVs and lysosomes could be a determinant of PV enlargement, as the depletion of secondary lysosomes [25] and acidic vesicles, as shown in this report, is observed during parasite establishment. Additionally, the up-regulation of host cell lipid biosynthesis triggered by the parasite [28] could increase the repertoire of membrane donors to *Leishmania* PVs development. This up-regulation was not observed in the analysis of gene expression of macrophages infected with *L. major* promastigotes [29].

After lysosomal marker acquisition (by means of fusion with acidic vesicles, lysosomes and/or late endosomes), the membrane input into *Leishmania* PVs and their stability as large compartments could be related to continuous fusion with ER-derived or Golgi-derived vesicles [1], [3] instead of fusion with secondary lysosomes, late endosomes, or vicinal *L. amazonensis* PVs.

The homotypic fusion between *L. amazonensis* PVs was described by Real and colleagues and occurs mainly after 24–48 h of intracellular infection [16]. Fusion between PVs before 12 h of infection was seldom observed. Indeed, Lippuner and colleagues found that a minority of Rab5-positive *L. mexicana* PVs fuse with each other in the first hours of intracellular infection [30]. The fusion between *L. amazonensis* PVs did not contribute to sustain PV enlargement because fused PVs regained their dimensions, a process probably related to compensatory membrane recycling. Although the results suggest a selective nature for PV membrane composition, the host cell/parasite components retained by large PVs to maintain their homeostasis remain to be elucidated. The output of membrane from large PVs could be incorporated by the plasma membrane (displaying parasite proteins on host cell surface) and other cytoplasmic organelles such as the ER.

The spacious PV represents a strategy to subvert host cell defenses, providing an environment with lower activities and/or concentrations of hydrolytic enzymes [9], [31]–[33]. Considering that *Leishmania* from the *L. mexicana* complex are resistant to IFN-γ–mediated macrophage activation and high NO concentrations [34], the unique morphology of large vacuoles could be vital for the establishment of these parasites. Recent studies documented that *L. major* procyclic promastigotes survive and multiply within *L. amazonensis* PVs after interspecific PV fusion in doubly infected macrophages [17]. As expected, the procyclic promastigotes were destroyed within their own phagolysosomes but were spared from destruction once they entered the spacious *L. amazonensis* PVs.

*L. major*, the first organism in the genus to have its genome sequenced [35], is one of several *Leishmania* species in which amastigotes develop tight PVs that undergo fission during parasite multiplication in host cells. These amastigotes are generally individualized in PVs that maintain their dimensions and possibly require different strategies to avoid host cell defenses. PV fission is displayed by several important pathogens that live in vacuoles throughout their intracellular life cycle, such as *Leishmania* and *Mycobacterium* [36]. The process is likely to require the mobilization of membrane sources because replicating amastigotes require increases in PV dimensions prior to division, which implies an increase in PV membrane surface area and volume.

After parasite division, there is an intermediary state of double-occupancy PVs in which a vacuolar interface between recently-divided parasites and the PV membrane is visible using lysosomotropic probes. This detectable vacuolar space is polarized in agreement with the polarized event of amastigote division, which suggests a period of hours in which two dividing *L. major* amastigotes share the same PV with a small, acidic lumen. Indeed, in the late 1970s, a Thorotrast-rich vacuolar space located between two dividing *L. major* amastigotes was documented by electron microscopy [23]. Amastigote division within tight PVs could cause increase in PV fusogenicity with small vesicles by modifying the membrane curvature, which could physically assist SNARE machinery for membrane fusion [37]. An alternative interpretation of the Lysotracker clusters on dividing *L. major* amastigotes is that acidic vesicles could be mobilized to PV membrane sites in hotspots, where membrane input preferentially occurs.

The numeric decrease of host acidic vesicles during *L. major* PV fission or *L. amazonensis* PV enlargement is likely related to a higher demand of host membrane sources for the biogenesis of *Leishmania* PVs, at least in the first 4 days of intracellular infection. The host cell reservoirs of acidic vesicles would partially account for membrane incorporation of fission-prone *L. major* PV and partition in two new PVs or massive incorporation of vesicles into enlarging *L. amazonensis* PVs. Considering the membrane surface area of *Leishmania* PVs, a large *L. amazonensis* PV with 20 µm of diameter has the approximate surface area of 16 *L. major* tight-fitting PVs, indicating that *Leishmania* PVs would require approximate amounts of host cell membrane for their biogenesis regardless of the different PV architectures (tight-fitting vs. loose vacuoles). The contribution of each acidic vesicle to PV biogenesis may be also hypothesized: approximately 50 detected acidic vesicles (1 µm in diameter) in macrophages infected with *L. major* or *L. amazonensis* are consumed in the course of intracellular infection. This represents a hypothetical volume contribution of 30 µm^3^ to *Leishmania* PVs, which only partially accounts for *L. major* or *L. amazonensis* PV dimensional doubling.

Indeed, other mechanisms of *Leishmania* PV volume control were addressed in the literature and include fusion with ER vesicles, acquisition of water and ion transport channels, and parasite secretion of exosomes and macromolecules inserted in PV membranes, or displayed in parasite membrane surfaces [3], [33], [38]–[41]. Additionally, *Leishmania* can internalize PV membrane components: in a process resembling host membrane “clearance,” *L. amazonensis* can actively internalize and digest MHC class II, possibly by its posterior pole that interacts with PV membranes [42]. This process could also be conserved in *Leishmania* with membrane-bound PVs participating in the control of membrane input into the PVs. The polarization of lysotracker clusters in dividing *L. major* parasites suggests that parasite poles could also participate in the PV biogenesis of tight-fitting phenotype.

Although *L. major* PVs incorporate phagolysosomal markers on their membranes, they were unable to retain amounts of lysosomotropic content probes comparable to other tight-fitting phagosomes (i.e. latex beads or aldehyde-fixed amastigotes). A less acidic environment could account for Lysotracker-negative *L. major* PVs, which could present lower abundance of vesicular proton ATPases than *L. amazonensis* PVs. Osorio y Fortea and colleagues [28] showed that eight isoforms of vesicular proton ATPases subunits are up-regulated in macrophages infected with *L. amazonensis* amastigotes; in contrast, Gregory and colleagues [29] showed that only one isoform of these ATPases, V1 subunit H, is up-regulated in macrophages infected with *L. major* promastigotes. In these same experiments, a V0 subunit A2 is down-regulated and other lysosomal components (such as two isoforms of a lysosomal-associated transmembrane protein 5 and an isoform of acid phosphatase 2) are down-regulated. Non-acidic phagosomes displaying phagolysosomal markers on their membranes were associated with exclusion of these proton ATPases in *Mycobacterium* phagosomes [43]. It is possible that *L. major* PVs maintain the exclusion of vesicular proton ATPases for amastigote replication, in a process similar to what occurs in *L. donovani* promastigote PVs [44].

By presenting these fundamental pre-mechanistic data obtained by live imaging, we highlighted that *Leishmania* PVs are dynamic structures that remodel their shapes, allowing these structures to develop a privileged intracellular niche where *Leishmania* parasites survive and multiply. Several pathogens can utilize this process; describing and understanding the nature of these vacuoles in live infected cells are crucial steps towards the understanding of how parasites evade host immune responses [45].

## Supporting Information

Video S1 Enlargement of *L. amazonensis* PVs. Live imaging of *L. amazonensis* PVs loaded with Lysotracker (green) in infected macrophages. Image acquisition started after 2 h (A) and 96 h (B) of intracellular infection. The time of image acquisition is shown as d:hh:mm.
(MOV)

Video S2 Volume and diameter measurements of *L. amazonensis* PVs using isosurfaces. (A) Lysotracker signal exhibited by three large PVs in the same infected macrophage during the course of image acquisition. (B) Software recognition of the same three PVs. (C–D) For each recognized PV, the software attributed an isosurface that permitted PV volume, diameter, and Lysotracker RFI measurements. (C) PV diameter was measured at an intermediary plane of the multidimensional image. (D) PVs are represented by a statistic-coded isosurface for which color is in accordance with volume measurements, ranging from cyan (smaller volume) to magenta (larger volume). Multidimensional acquisition started after 2 h of infection, and the time of acquisition is shown as d:hh:mm.
(MOV)

Video S3 Macrophage acidic vesicles decrease in number during *L. amazonensis* PV biogenesis and growth. (A–C) Separation of acidic vesicles from *Leishmania* PVs using the same fluorescence channel. (A) Multidimensional image of macrophages infected with *L. amazonensis* and loaded with Lysotracker. A window for software analysis was established to identify acidic vesicles exclusively in the selected macrophage. (B) Isospots 1 µm in diameter were attributed to Lysotracker channel voxels in multidimensional images. The software identified small vesicles surrounding *L. amazonensis* PVs but interpreted PVs as clusters of Lysotracker-positive vesicles. Each isospot has a statistic-based color corresponding to the mean Lysotracker RFI (colored bar). (C) By adjusting the thresholds of isospots detection based on Lysotracker RFI, the software can attribute isospots to the acidic vesicles (orange isospots in the second image, excluding the fluorescence signal of *Leishmania* PVs. Multidimensional acquisition started after 2 h of infection, and the time of acquisition is shown as hh:mm.
(MOV)

Video S4 Fusion and remodeling of *L. amazonensis* PVs. (A) Multidimensional imaging of *L. amazonensis* PVs loaded with Lysotracker in live infected macrophages. In the image, Lysotracker fluorescence of an infected macrophage containing four PVs – two of them fuse at 0d23:45. (B) Isosurfaces representative of each PV in the multidimensional image. Isosurfaces have statistic-based color according to their measured volumes ranging from cyan (smaller volume) to magenta (larger volume). Image acquisition started after 48 h of infection, and the time of acquisition is shown as d:hh:mm.
(MOV)

Video S5 Replication of *L. major* amastigotes in tight-fitting PVs. (A) Multiplication of *L. major*-DsRed2 amastigotes recorded by fluorescent time-lapse microscopy of infected macrophage cultures. The record comprises the period in which the parasite population doubles. Time-lapse acquisition started after 2 h of infection, and the time of acquisition is shown as d:hh:mm. (B) Multidimensional image of live or dead (fixed) *L. major*-DsRed2 amastigotes – indicated on the video, internalized by macrophages loaded with Lysotracker or FITC-dextran lysosomotropic probes. Lysotracker or FITC-dextran clusters are commonly detected on an amastigote pole before division. Acquisition started after 2 h of infection, and the time of acquisition is shown as d:hh:mm.
(MOV)

Video S6 Fission of *L. major* PVs. (A) Fission inferred by differences in Lysotracker RFIs surrounding dividing parasites. Multidimensional imaging of dividing *L. major*-DsRed2 amastigotes (red) hosted by a macrophage loaded with Lysotracker (green). Isosurfaces were attributed to replicating amastigotes, allowing the measurement of Lysotracker RFI surrounding parasites. The amastigotes remain next to each other after replication and the vacuolar interface between them is observed at the initial time points. An increase in the Lysotracker signal was detected in one dividing amastigote, whereas the other remained in a PV with a low Lysotracker signal. By the end of the recordings, the amastigotes were surrounded by Lysotracker at low intensities, and no Lysotracker-positive vacuolar interface was observed between them. Image acquisition started after 48 h of infection, and the time of acquisition is shown as hh:mm. (B) *L. major* PV fission observed in infected RAW 264.7 macrophages expressing LAMP1 and Rab7 tagged with GFP. A double-occupancy PV precedes the formation of individualized *L. major* PVs, between time points 10:40 and 12:31. Fission is completed after the incorporation of phagolysosomal-membrane in the interface between dividing parasites (time point 12:31). Image acquisition started after 3 h of infection, and the time of acquisition is shown as dhh:mm.
(MOV)

Video S7 Double-occupancy *L. major* PVs present dynamic lysotracker clusters in the interface between amastigotes. (A) Multidimensional imaging of dividing *L. major*-DsRed2 amastigotes (red) hosted by a macrophage loaded with Lysotracker (green). Polarized Lysotracker clusters are observed in amastigotes prior to division; after division, clusters are found between two divided parasites. Image acquisition started after 3 h of infection, and the time of acquisition is shown as d:hh:mm. (B–C) The Lysotracker cluster between dividing parasites was observed in multidimensional images. The attribution of an isosurface to the Lysotracker-positive interface permitted the measurement of its volume at each time point. The isosurface contains statistic-based color information ranging from cyan (smaller volumes) to magenta (larger volumes). Image acquisition started after 2 h of infection, and the time of acquisition is shown as d:hh:mm. (D–E) Another multidimensional image acquired in shorter time intervals, showing the lysotracker-positive interface between dividing parasites. Image acquisition started after 48 h of infection, and the time of acquisition is shown as hh:mm.
(MOV)

Video S8 Macrophage acidic vesicles decrease in number during *L. major* replication. (A) Multidimensional imaging of dividing *L. major*-DsRed2 amastigotes (red) hosted by a macrophage loaded with Lysotracker (green). The record comprises the period in which the parasite population doubles. (B) The software attributed to parasites an analytical isosurface (gray), allowing the measurement of Lysotracker RFIs of voxels surrounding amastigotes. Additionally, isospots were attributed to macrophage acidic vesicles (green), quantified in the course of multidimensional imaging. Image acquisition started after 24 h of infection, and the time of acquisition is shown as d:hh:mm.
(MOV)
